# Geotechnical Characterization of Mined Clay from Appalachian Ohio: Challenges and Implications for the Clay Mining Industry

**DOI:** 10.3390/ijerph8072640

**Published:** 2011-06-28

**Authors:** Anthony R. Moran, Hiroshan Hettiarachchi

**Affiliations:** 1 CH2M Hill, 7927 Nemco Way, Suite 120, Brighton, MI 48116, USA; E-Mail: Anthony.Moran@ch2m.com; 2 Department of Civil Engineering, Lawrence Technological University, 21000 West Ten Mile Road, Southfield, MI 48075, USA

**Keywords:** mined clay, shale, Appalachian Ohio, clay content, grain size distribution, sieve, analysis, hydrometer analysis, Atterberg limits, hydraulic conductivity, Recompated Soil Liners (RSL)

## Abstract

Clayey soil found in coal mines in Appalachian Ohio is often sold to landfills for constructing Recompacted Soil Liners (RSL) in landfills. Since clayey soils possess low hydraulic conductivity, the suitability of mined clay for RSL in Ohio is first assessed by determining its clay content. When soil samples are tested in a laboratory, the same engineering properties are typically expected for the soils originated from the same source, provided that the testing techniques applied are standard, but mined clay from Appalachian Ohio has shown drastic differences in particle size distribution depending on the sampling and/or laboratory processing methods. Sometimes more than a 10 percent decrease in the clay content is observed in the samples collected at the stockpiles, compared to those collected through reverse circulation drilling. This discrepancy poses a challenge to geotechnical engineers who work on the prequalification process of RSL material as it can result in misleading estimates of the hydraulic conductivity of the samples. This paper describes a laboratory investigation conducted on mined clay from Appalachian Ohio to determine how and why the standard sampling and/or processing methods can affect the grain-size distributions. The variation in the clay content was determined to be due to heavy concentrations of shale fragments in the clayey soils. It was also concluded that, in order to obtain reliable grain size distributions from the samples collected at a stockpile of mined clay, the material needs to be processed using a soil grinder. Otherwise, the samples should be collected through drilling.

## 1. Introduction

Clayey soil found beneath a coal layer, which is also known as underclay, is an ideal construction material for Recompacted Soil Liners (RSLs) in landfills due to its low hydraulic conductivity and relatively low cost. Coal mines in Appalachian Ohio typically encounter large volumes of underclay due to the depositional nature of coal formations there. Clay mines make a profit and increase usable space at the mines by mining and selling the underclay to landfills that do not have sufficient clay borrow sources located within their property boundaries or permitted limits of waste. Landfills can save money utilizing the mined clay if the haul distance is short enough with respect to the expense of using additional geosynthetic materials.

The research described in this paper comes to light from experiences with underclay from Appalachian Ohio, which has shown some unique characteristics in its composition. The Ohio region, at the base of the Appalachian basin, is comprised of shale enriched clay deposits found beneath coal layers [1]. These clay deposits are classified with a layer number. The layering system is based on the cyclothemic depositions of the coal layers in this region. The deeper the clayey soil layer, the lower the number identification for the layer [1].

The solid waste division of the Ohio Environmental Protection Agency (OEPA) requires that RSL material be pre-qualified prior to use in construction [2]. This means that samples are analyzed for grain-size distribution, Atterberg limits, compaction characteristics and hydraulic conductivity prior to their acceptance for use. For hydraulic conductivity, remolded samples satisfy the OEPA requirement for the establishment of the RSL’s hydraulic conductivity [2]. Clay mines typically pay for the cost of the drilling exploration and allow consultants onto their sites to monitor the drilling of potential RSL borrow sources. The method of collecting samples for laboratory testing through drilling as well as collecting samples from representative stockpiles are deemed acceptable.

Standard field sampling methods and laboratory sample preparation procedures, should not produce different grain-size distributions for the same material. An engineering mind would expect that a sample collected within the borrow location at a clay mine would classify the same and contain the same clay content as when the soils are excavated and hauled to a stockpile at a landfill and re-tested. But experience with the mined clay from Appalachian Ohio has revealed that this is not always the case, and the mined clay from Appalachian Ohio has shown drastic differences in particle size distribution depending on the sampling and/or laboratory processing methods. More particularly, the clay content of the material, *i.e.* particles finer than 0.002 mm as per AASHTO [3], has been shown to vary substantially. The research described in this paper was conducted to investigate how the sampling and/or laboratory processing methods could affect the grain-size distribution of mined clay from Appalachian Ohio.

## 2. Materials and Methods

Underclay samples were collected from a location in Appalachian Ohio that was previously used for coal mining. This underlay layer was specifically identified as Number 5 clay [1]. The clay source was originally under approximately 60 feet of overburden.

### 2.1. Sample Collection

**Figure 1:**
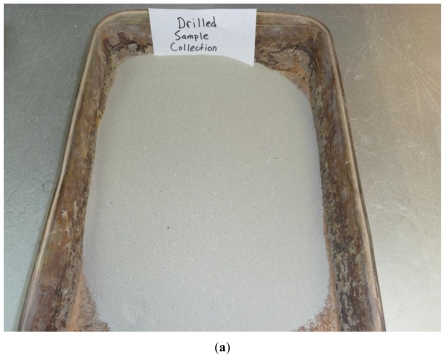

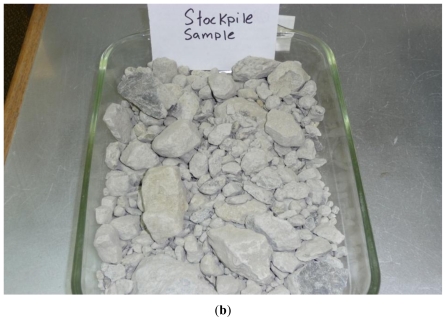
(**a**) Samples collected through drilling (D-samples); (**b**) Samples collected at the stockpiles (S-samples).

About 100 soil samples were collected at different depths in the clayey soil deposit using a hollow stem auger with reverse circulation drilling. Before transporting to the laboratory, soil samples were first visually classified in the field and then collected in one gallon ziplock bags and labeled in numerical order by collection. Samples retrieved by this method were observed to have flour-like consistency as can be seen in Figure 1(a). All soil samples collected by drilling visually appeared nearly identical. The drilling efforts spanned approximately three days at the proposed borrow location at the clay mine. The samples collected through reverse circulation drilling were designated as D-samples during this research.

Once the clayey soil was mined and stockpiled, a second set of samples were collected from the stockpiles. They were designated as S-samples to indicate the method of collection. During stockpiling, accurate records were kept so that the original location and the depth of the soil could be easily identified to compare S-samples with previously collected samples D-samples. Depth and location identification was conducted by utilizing a hand held GPS unit. When the area of the clay mine was excavated, the approximate location of this material was flagged in the stockpiles. By doing this, it was ensured that the S-soil samples were also from the same locations where the D-samples were collected. S-samples were collected and transported in five gallon buckets.

About 65 S-samples were finally collected to compare with D-samples. Samples retrieved from this method did not have a fine consistency as the D-samples, and instead the S-samples contained many clay clods, as seen in Figure 1(b).

### 2.2. Sample Processing

For the initial soil characterization, soil samples were processed either completely manually or using a soil processor. During manual processing, soil samples were first allowed to air dry at room temperature then placed over a #4 screen and pushed through by hand. This manual preparation was designated as M-processing.

During sample preparation using the soil processor, air dried samples were first placed in the soil processor. Then the weighted lid of this machine applied a normal dead load to the sample as an air-operated arm moves the #4 screen perpendicular to the sample. The results produced by this method were more or less identical to the soil samples produced by the above process and hence this preparation method was also designated as M-processing.

**Figure 2:**
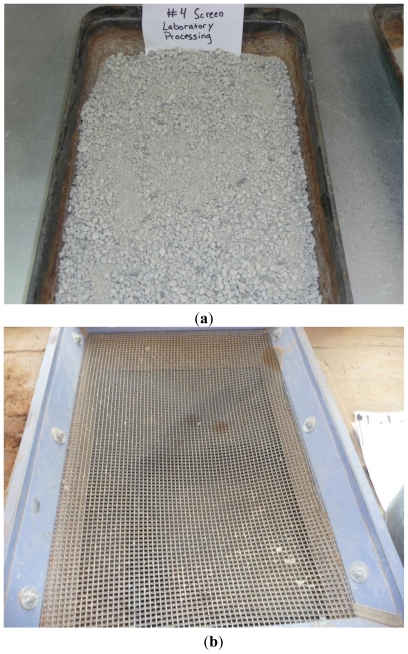
(**a**) A stockpile sample processed manually using #4 screen (SM) (**b**) #4 screen used in manual processing.

D-samples did not need much of a preparation other than air drying as they were already fine in consistency. However, to make the research process consistent, D-samples were also nominally processed manually by sieving through the #4 screen. Soil clods in S-samples needed to be processed before testing. An example of a manually processed S-sample is shown in Figure 2(a). The #4 screen used in M-processing can be seen in Figure 2(b).

### 2.3. Geotechnical Characterization

ASTM test protocols were followed during geotechnical testing. To characterize the material, particle size analysis (ASTM D421 and D422), Atterberg limits (ASTM D4318), classification (ASTM D2487), modified Proctor (ASTM D1557) and hydraulic conductivity (ASTM D5084) tests were carried out as per the ASTM test protocols [4]. Hydraulic conductivity tests were slightly modified to suit the requirements of this research. Before testing for hydraulic conductivity, the samples were placed in a sealed container to temper for at least 16 hours. Then samples were remolded (mold dimensions: 2.79″ diameter × 3″ height) and compacted to 90 percent of the maximum dry unit weight, as determined by the modified Proctor test. Remolded specimen was then placed within a flexible wall permeameter cell.

## 3. Results, Analysis and Discussion

All D-samples and S-samples were first processed manually (hence designated as DM and SM) and subjected to mechanical analysis to obtain their gradation.

**Figure 3:**
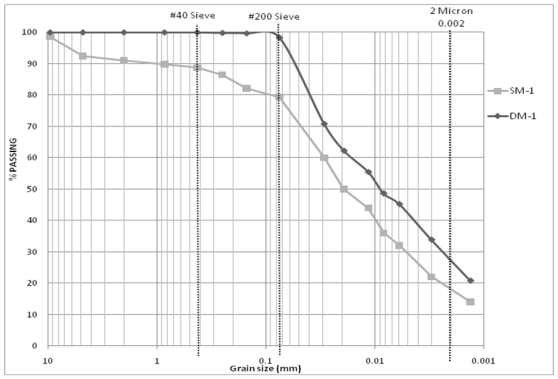
Comparison of grain size distributions: manually processed drilled sample (DM-1) and manually processed sample collected at the stockpile (SM-1).

Size distributions of all theses samples are given in Moran [5]. Based on the results of mechanical analysis, 13 DM-samples with similar clay content (approximately 28%) were selected for further analysis. Size distributions of these 13 DM-samples are given in Table 1. SM-samples, those were originated from the same locations as in DM-samples were also selected for further processing. Size distributions of the 13 SM-samples are given in Table 2. As is evident from Tables 1 and 2, the grain size distributions of DM-samples do not match with the grain size distributions for SM-samples, though they originated from the same locations. The difference in clay content was nearly 10 percent. To illustrate this difference in clay content, grain-size distributions for DM-1 and SM-1 are compared in Figure 3.

Both DM and SM soil samples were then subjected to further geotechnical characterization through Atterberg limits, classification per USCS, modified Proctor and hydraulic conductivity tests. According to the results of these tests summarized in Table 1 and Table 2, the material is rather homogenous. Hydraulic conductivity is typically a function of the clay content. But the 10% difference in the clay content has not even made any considerable impact on hydraulic conductivity. Therefore it is clear that the clay content of one of the sample types is incorrect.

### 3.1. Sample Processing by a Soil Grinder

**Figure 4:**
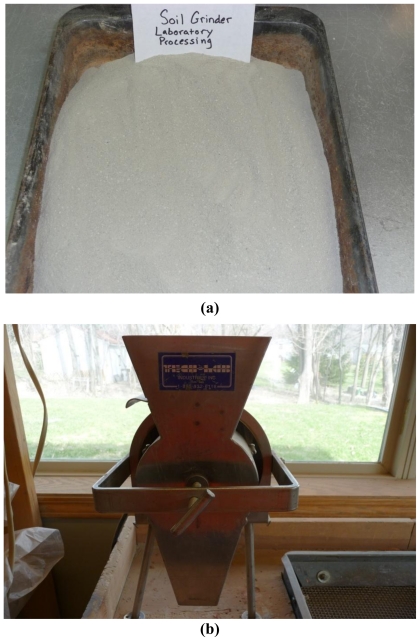
(**a**) A stockpiled sample processed through grinding (SG); (**b**) the soil grinder used for processing.

During the next phase of the research, the laboratory processing of S-samples were performed using a soil grinder (hence SG-samples). The mechanical soil grinder utilized is the one typically used for preparing samples for Atterberg limits and specific gravity tests. Air dried samples were placed into the soil grinder which consists of an electric motor driving a ½ inch shaft with ¼ inch spinning steel bars. The grinder “broke the soil down” to clods to the approximate size of a number 10 sieve or smaller. This method was designated as G-processing (hence processed samples designated as SG-samples). An example of a sample prepared by this method is shown in Figure 4(a) and the apparatus for grinding can be seen in Figure 4(b). Visual appearance of the ground samples appeared similar to the samples collected through the reverse circulation drilling exploration (D-samples).

**Figure 5:**
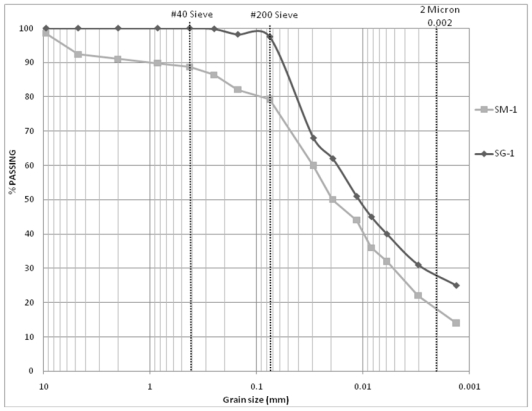
Comparison of grain size distributions: manually processed stockpiled sample (SM-1) *vs*. grinder processed stockpiled sample (SG-1).

The results of particle size analysis, Atterberg limits, modified Proctor and hydraulic conductivity tests conducted on the SG-samples are displayed in Table 3. Figure 5 compares the grain-size distributions for SM-1 and SG-1 samples (from the same location). The plot shows a clear difference in the clay content in the gradation curves obtained from manual processing and grinder processing of the same S-samples.

**Figure 6:**
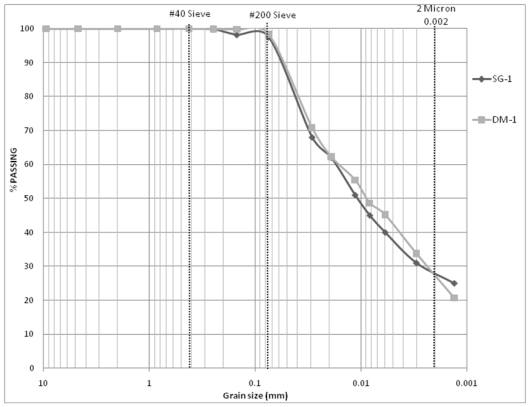
Comparison of grain size distributions: Manually processed drilled *vs*. grinder processed stockpiled samples (DM-1 & SG-1).

However, referring back to Tables 1–3, little to no change is observed in other geotechnical characteristics. The small variations noted are well within the typical expected test margins of error for Atterberg limits, modified Proctor and hydraulic conductivity tests. On the other hand, the grain size distributions of SG-samples (Table 3) nearly mimic the results for the DM-samples (Table 1). Figure 6 compares the grain-size distributions of DM-1 and SG-1 samples.

The results of the laboratory samples processed using a soil grinder, indicate the impact of the mechanical process to have a direct affect on the grain-size distribution. Since the same S-samples showed a clay content similar to that of DM when processed using a grinder (SG), it is clear that the discrepancy could be due to the soil clods that are not easily broken during processing. Standard ASTM test protocols for sieve and hydrometer tests have been in use for some time and they have an excellent track record for producing repeatable and reliable data. The question that needs an answer is that why mined clay samples collected at the stockpiles have become an exception.

### 3.2. Stockpile Mined Clay Samples from Other Locations in the Midwest

To see if the use of a soil grinder has any impact on the grain size distribution, comparable clayey soil samples obtained from two other locations in the Midwest were analyzed. Four samples from southwestern Michigan and another four from central Kentucky were used for this purpose. Samples were split in half prior to processing. One set of samples was first processed by hand through a number 4 screen. The other set of samples was then processed using the soil grinder after air drying. Both sets were then subjected to gradation analysis, Atterberg limits, modified Proctor, and hydraulic conductivity testing.

**Figure 7:**
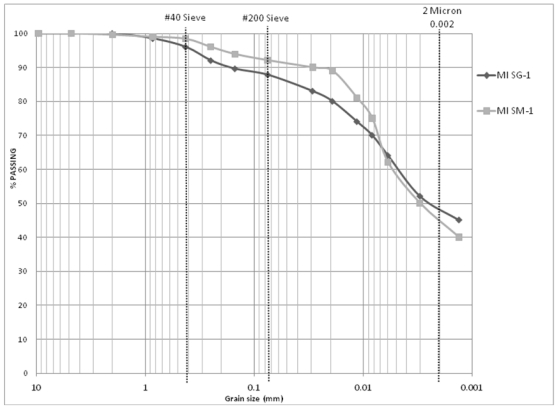
Comparison of grain size distributions: manually processed *vs*. grinder processed stockpiled samples of mined clay from Southwest Michigan (MI SM-1 & MI SG-1).

**Figure 8:**
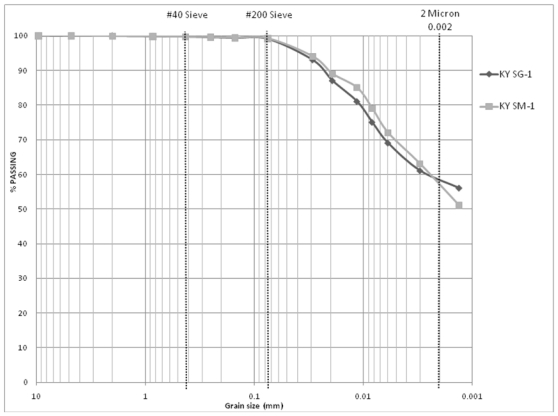
Comparison of grain size distributions: manually processed *vs*. grinder processed stockpiled samples of mined clay from Central Kentucky (KY SM-1 & KY SG-1).

Included in Table 4 and Table 5 are the results from the tests conducted on samples from southwestern Michigan and central Kentucky respectively. No significant change is noted in the Atterberg limits, modified Proctor, hydraulic conductivity as well as the gradation of the test specimen based on the processing method. Figure 7 compares the gradation curves of the mined clay from southwestern Michigan collected at the stockpiles but processed manually (MI SM-1) and using a soil grinder (MI SG-1). Figure 8 presents the same comparison for the samples from Central Kentucky (KY SM-1 and KY SG-1). Figure 7 and Figure 8 provide examples of how well the gradation curves from two methods compare with each other. The conclusion these results provide is that the discrepancy observed in mined clay from Appalachian Ohio is an exception and may not be due to a deficiency of the standard testing methods.

### 3.3. Geological Background of Clay Deposits in Appalachian Basin

Information on the regional geology and the composition of mined clay from Appalachian Ohio was taken into consideration to investigate the discrepancies it shown in the grain size analysis. As explained before, soil found beneath coal layers at the base of the Appalachian basin is comprised of shale enriched clay deposits [1]. Shale in this region is mostly of lightly indurated silt and clay that can be broken with little force applied with the addition of moisture. The shale, depending on moisture content, will either appear as a gravel sized particle or as a clay clod. With the cyclothemic affects in Appalachian Ohio, one would expect a large variety and discontinuity within and between the sedimentary layers. Natural moisture content makes a direct influence on the visual classification of this soil. The higher the natural moisture content, the more likely the material will be visually classified as clayey soil and not as shale.

This specific composition of mined clay from Appalachian Ohio reveals that the soil clods observed in the stockpile samples are mostly the fragments of shale. Manual processing does not break all shale clods. The dispersing agent used during the hydrometer test is only able to separate some of the fines into individual particles, but not strong enough to break the shale fragments into the respective parent materials (*i.e*. clay and silt). Therefore, it is not surprising to see a lower clay fraction in SM-samples, because the small shale fragments may have behaved similar to coarse grained soils during the mechanical analysis. When a grinder was used to process soils, it was able to break the shale fragments into smaller clods. These smaller clods must have allowed the dispersing agent to more readily separate the shale fragments into its parent particles and increase the clay fraction in SG-samples. This explains why SG-samples behaved similar to DM-samples during mechanical analysis (Figure 6).

On the other hand, the mined clay from southwestern Michigan was from a glacial deposit and samples from central Kentucky were from a cyclothemic deposit. These samples from Michigan and Kentucky were high in clay content with traces of sand. Unlike the soil samples from Appalachian Ohio, none of these other samples would be considered to contain any shale fragments. As a result, they did not exhibit a considerable difference in the grain size distributions based on the processing method. Therefore, the presence of shale fragments should be the primary cause for the discrepancies observed in the grain-size analysis.

### 3.4. Impact on Clay Mining Industry

**Figure 9:**
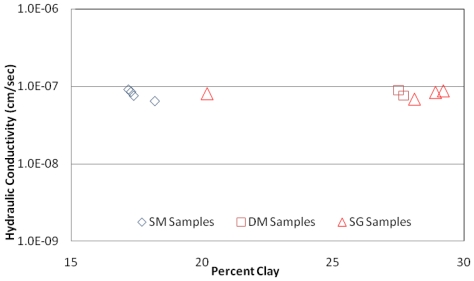
Hydraulic conductivity *vs*. percent clay content obtained for DM, SM and SG samples.

Hydraulic conductivity is the most important characteristic of RSL material and The State of Ohio has used 20 percent as a requirement for borrow soils used in RSL systems [2]. As per the results shown in Table 1 through Table 5, SM samples do not meet the 20% clay content requirement, but SG and DM-samples do meet this requirement. However, it is interesting to note that all three types of samples are from the same origin and they share the same hydraulic conductivity. Hydraulic conductivity values obtained for all three types of samples are compared in Figure 9. As can be seen in Figure 9 all hydraulic conductivity values are bordering along 1.0 × 10^−7^ cm/sec regardless of the sample collection/processing method.

When soil samples are delivered to a geotechnical laboratory for testing, geological history, sampling methods, and the expectations of soil evaluation results are seldom provided to the laboratory staff. The geotechnical laboratory is typically expected to act as an independent third party in the soil evaluation process even if the laboratory is within the same company as the sampling technician. This independent behavior works well when the testing standards prescribed within the industry include limited error margins. However, this intentional lack of communication between the geotechnical laboratory and the geotechnical engineer can cause problems when soils with unique characteristics such as the mined clay from Appalachian Ohio are tested.

If not instructed specifically, a laboratory may or may not use a grinder for processing soils, and depending on that decision, even a suitable soil may very well be rejected. It was reported that in 2003, over 2.1 million tons of clay were mined in Ohio [6]. With prices averaging $20/ton, selling mined clay for RSL construction is significant to Ohio’s mining industry. Therefore, decision making purely based on the apparent clay content without paying much attention to the hydraulic conductivity, can make a considerable negative impact on the clay mining industry.

## 4. Conclusions

The following conclusions were from the material discussed in this research. It should be noted that all conclusions are exclusively applicable to mined clay from Appalachian Ohio and may not be applicable to other general type of clayey soils.

- Grain size distributions of mined clay from Appalachian Ohio exhibit sensitivity to sampling and/or laboratory processing methods. Other geotechnical characteristics such as Atterberg limits, compaction (based on modified Proctor method) and hydraulic conductivity were not sensitive to sampling and or laboratory processing methods.
- The Presence of shale fragments in mine clay from Appalachian Ohio was found to be the reason for observing sampling and laboratory processing dependant results in particle size analysis of mined clay from Appalachian Ohio.
- Manual processing is not sufficient to separate shale fragments into individual silt and clay particles in the soil samples collected at mined clay stockpiles. Samples collected through drilling or samples collected at the stockpiles but processed using a soil grinder, seem to be producing more reliable grain size distribution data for mined clay from Appalachian Ohio.
- Use of clay content as the sole criteria to accept or reject Mined clay from Appalachian Ohio for RSL applications could produce misleading results. If clay content is the only criteria to be used, the samples for mechanical analysis should be collected through drilling or else samples collected at stockpiles should be processed using soil grinder.

## Figures and Tables

**Table 1:** Geotechnical characteristics of DM-samples selected for further processing.

Sample Number	Grain Size Distribution						Atterberg			Uscs Soil Type	Compaction Characteristics Based On Modified Proctor Test^2^		Hydraulic Conductivity (cm/sec)

	% Finer	% Finer	% Finer	% Finer	% Finer	% Finer	Limits^1^						
	12.5 mm	9.5 mm	#4	#40	#200	.002 mm	LL	PL	PI		γ_dmax_ (kN/m^3^)	OMC (%)	
DM-1	100.0	100.0	100.0	100.0	98.3	27.5	30	16	14	CL	–	–	–
DM-2	100.0	100.0	100.0	82.1	69.4	27.7	29	16	13	CL	21.2	6.6	7.6 × 10^−8^
DM-3	100.0	100.0	100.0	99.5	93.2	27.5	27	17	10	CL	22.0	7.1	9.0 × 10^−8^
DM-4	100.0	100.0	100.0	94.9	83.2	28.3	28	15	13	CL	–	–	–
DM-5	100.0	100.0	100.0	99.7	96.1	28.1	24	14	10	CL	–	–	–
DM-6	100.0	100.0	100.0	98.9	92.6	28.8	23	15	8	CL	–	–	–
DM-7	100.0	100.0	100.0	83.3	73.3	29.8	26	16	10	CL	–	–	–
DM-8	100.0	100.0	100.0	96.5	89.1	27.7	28	16	12	CL	–	–	–
DM-9	100.0	100.0	100.0	98.9	94.6	27.2	28	18	10	CL	–	–	–
DM-10	100.0	100.0	100.0	80.7	74.4	27.7	27	17	10	CL	–	–	–
DM-11	100.0	100.0	100.0	98.9	95.9	27.6	33	19	14	CL	–	–	–
DM-12	100.0	100.0	100.0	99.8	95.4	28.2	25	17	8	CL	–	–	–
DM-13	100.0	100.0	100.0	99.8	93.8	27.6	26	17	9	CL	–	–	–

1 LL = liquid limit, PL = plastic limit, PI = plasticity index;

2 γ_dmax_ = maximum dry unit weight, OMC = optimum moisture content.

**Table 2:** Geotechnical characteristics of SM-samples selected for further processing.

Sample Number	Grain Size Distribution						Atterberg			Uscs Soil Type	Compaction Characteristics Based On Modified Proctor Test^2^		Hydraulic Conductivity (cm/sec)

	% Finer	% Finer	% Finer	% Finer	% Finer	% Finer	Limits^1^						
	12.5 mm	9.5 mm	#4	#40	#200	.002 mm	LL	PL	PI		γ_dmax_ (kN/m^3^)	OMC (%)	
SM-1	100.0	98.5	92.4	88.7	79.3	17.3	30	17	13	CL	21.9	7.3	**8.5 × 10**^−^**^8^**
SM-2	100.0	100.0	94.6	87.8	75.7	17.4	28	18	10	CL	21.3	5.9	7.6 × 10^−8^
SM-3	100.0	100.0	95.5	87.1	76.9	17.2	28	18	10	CL	21.9	8.0	9.1 × 10^−8^
SM-4	100.0	100.0	92.5	91.8	83.1	18.2	29	17	12	CL	21.7	7.0	6.5 × 10^−8^
SM-5	100.0	100.0	98.7	94.6	85.6	17.3	29	18	11	CL	–	–	–
SM-6	100.0	99.4	96.0	89.0	78.1	18.5	24	16	8	CL	–	–	–
SM-7	100.0	100.0	99.5	93.9	83.7	20.6	27	17	10	CL	–	–	–
SM-8	100.0	99.2	94.1	89.2	79.0	18.8	28	16	12	CL	–	–	–
SM-9	100.0	100.0	95.6	93.2	80.2	19.5	28	19	9	CL	–	–	–
SM-10	100.0	99.2	97.5	90.7	78.6	19.3	28	17	11	CL	–	–	–
SM-11	100.0	100.0	100.0	92.8	82.4	19.1	31	19	12	CL	–	–	–
SM-12	100.0	100.0	98.4	88.9	78.1	17.5	25	17	8	CL	–	–	–
SM-13	100.0	99.4	94.5	88.1	78.0	17.6	27	17	10	CL	–	–	–

1 LL = liquid limit, PL = plastic limit, PI = plasticity index;

2 γ_dmax_ = maximum dry unit weight, OMC = optimum moisture content.

**Table 3:** Geotechnical characteristics of SG-samples.

Sample Number	Grain Size Distribution						Atterberg			Uscs Soil Type	Compaction Characteristics Based On Modified Proctor Test^2^		Hydraulic Conductivity (cm/sec)

	% Finer	% Finer	% Finer	% Finer	% Finer	% Finer	Limits^1^						
	12.5 mm	9.5 mm	#4	#40	#200	.002 mm	LL	PL	PI		γ_dmax_ (kN/m^3^)	OMC (%)	
SG-1	100.0	100.0	100.0	100.0	97.5	28.9	29	15	14	CL	21.9	7.27	8.4 × 10^−8^
SG-2	100.0	100.0	100.0	90.0	80.0	20.2	27	19	8	CL	21.0	7.20	8.1 × 10^−8^
SG-3	100.0	100.0	100.0	98.4	92.4	29.2	27	18	9	CL	22.0	7.08	8.8 × 10^−8^
SG-4	100.0	100.0	100.0	91.8	89.5	28.1	28	18	10	CL	21.4	7.85	6.9 × 10^−8^
SG-5	100.0	100.0	100.0	96.5	95.4	29.1	29	18	11	CL	–	–	–
SG-6	100.0	100.0	100.0	98.4	92.0	28.0	25	15	10	CL	–	–	–
SG-7	100.0	100.0	100.0	96.1	88.7	25.0	26	16	10	CL	–	–	–
SG-8	100.0	100.0	100.0	97.2	94.5	27.5	29	15	14	CL	–	–	–
SG-9	100.0	100.0	100.0	96.4	95.2	27.4	29	15	14	CL	–	–	–
SG-10	100.0	100.0	100.0	98.5	94.2	29.5	30	18	12	CL	–	–	–
SG-11	100.0	100.0	100.0	96.5	91.0	26.0	31	20	11	CL	–	–	–
SG-12	100.0	100.0	100.0	91.0	82.5	22.4	26	16	10	CL	–	–	–
SG-13	100.0	100.0	100.0	99.1	96.9	29.2	28	17	11	CL	–	–	–

1 LL = liquid limit, PL = plastic limit, PI = plasticity index;

2 γ_dmax_ = maximum dry unit weight, OMC = optimum moisture content.

**Table 4:** Geotechnical characteristics of stockpiled mined clay-samples from Southwest Michigan processed manually and using a soil grinder.

Sample Number	Grain Size Distribution						Atterberg			Uscs Soil Type	Compaction Characteristics Based On Modified Proctor Test^2^		Hydraulic Conductivity (cm/sec)

	% Finer	% Finer	% Finer	% Finer	% Finer	% Finer	Limits^1^						
	12.5 mm	9.5 mm	#4	#40	#200	.002 mm	LL	PL	PI		γ_dmax_ (kN/m^3^)	OMC (%)	
MI SM-1	100.0	100.0	99.6	98.4	92.1	45.5	38	24	14	CL	18.8	12.45	6.8 × 10^−8^
MI SM-2	100.0	100.0	100.0	99.2	93.9	31.9	34	16	18	CL	19.1	12.36	5.4 × 10^−8^
MI SM-3	97.0	96.5	94.2	86.5	62.8	21.1	24	16	8	CL	–	–	–
MI SM-4	100.0	100.0	99.0	98.2	96.7	50.7	39	24	15	CL	–	–	–
MI SG-1	100.0	100.0	100.0	96.0	87.8	48.9	38	24	14	CL	18.6	12.55	7.0 × 10^−8^
MI SG-2	100.0	100.0	100.0	100.0	96.5	38.2	35	16	19	CL	19.0	12.44	5.3 × 10^−8^
MI SG-3	100.0	100.0	98.5	88.2	63.5	25.2	27	16	11	CL	–	–	–
MI SG-4	100.0	100.0	99.2	98.2	96.9	51.0	40	22	18	CL	–	–	–

1 LL = liquid limit, PL = plastic limit, PI = plasticity index;

2 γ_dmax_ = maximum dry unit weight, OMC = optimum moisture content.

**Table 5:** Geotechnical characteristics of stockpiled mined clay-samples from Central Kentucky processed manually and using a soil grinder.

Sample Number	Grain Size Distribution						Atterberg			Uscs Soil Type	Compaction Characteristics Based On Modified Proctor Test^2^		Hydraulic Conductivity (cm/sec)

	% Finer	% Finer	% Finer	% Finer	% Finer	% Finer	Limits^1^						
	12.5 mm	9.5 mm	#4	#40	#200	.002 mm	LL	PL	PI		γ_dmax_ (kN/m^3^)	OMC (%)	
KY SM-1	100.0	100.0	99.9	99.7	99.2	58.6	47	21	26	CL	18.0	15.27	3.2 × 10^−8^
KY SM-2	100.0	100.0	99.9	99.8	99.2	42.5	50	22	28	CH	18.5	13.89	4.1 × 10^−8^
KY SM-3	100.0	100.0	99.3	97.9	96.6	54.6	53	24	29	CH	–	–	–
KY SM-4	100.0	100.0	100.0	99.8	99.4	50.9	52	27	25	CH	–	–	–
KY GR-1	100.0	100.0	100.0	99.8	99.1	58.0	46	21	25	CL	18.3	14.91	3.4 × 10^−8^
KY GR-2	100.0	100.0	99.8	99.6	99.0	43.0	51	24	27	CH	18.5	14.01	4.1 × 10^−8^
KY GR-3	100.0	100.0	99.8	98.0	96.8	54.2	54	25	29	CH	–	–	–
KY GR-4	100.0	100.0	100.0	99.9	99.3	50.1	50	25	25	CH	–	–	–

1 LL = liquid limit, PL = plastic limit, PI = plasticity index;

2 γ_dmax_ = maximum dry unit weight, OMC = optimum moisture content.
